# Delayed Presentation of Porta Hepatis Injury Following Blunt Abdominal Trauma

**DOI:** 10.1155/1997/96710

**Published:** 1997

**Authors:** L. L. Lau, T. Diamond

**Affiliations:** Mater Hospital Crumlin Road Belfast BT14 6AB Ireland

## Abstract

A 73 year old lady developed abdominal pain, anaemia and obstructive jaundice 18 days after a road traffic accident. The jaundice was due to compression of the biliary confluence by a haematoma which was caused by a laceration of the left portal vein. The portal vein was repaired (lateral venorrhaphy) and post-operative recovery was uncomplicated. Porta hepatis injuries are difficult to diagnose and delayed presentation is not uncommon. Significant morbidity and mortality may ensue if aggressive management is not adopted.


HPB Surgery, 1997, Vol. 10, pp. 249–252
Reprints available directly from the publisher
Photocopying permitted by license only

© 1997 OPA (Overseas Publishers Association)
Amsterdam B.V. Published in The Netherlands
by Harwood Academic Publishers
Printed in India

*Case Report*

### Delayed Presentation of Porta Hepatis Injury Following Blunt Abdominal Trauma

L. L. LAU MB FRCS[a] and T. DIAMOND BSc MD FRCS FRCSI[b,*]

[a]*SHO in Surgery,* [b]*Consultant Surgeon; Mater Hospital Crumlin Road Belfast BT14 6AB Northern Ireland*

*(Received 7 December 1994)*

**A 73 year old lady developed abdominal pain, anaemia and obstructive jaundice 18 days after a road traffic accident. The jaundice was due to compression of the biliary confluence by a haematoma which was caused by a laceration of the left portal vein. The portal vein was repaired (lateral venorrhaphy) and post-operative recovery was uncomplicated. Porta hepatis injuries are difficult to diagnose and delayed presentation is not uncommon. Significant morbidity and mortality may ensue if aggressive management is not adopted.**

*Keywords:* Porta hepatis, portal vein, bile duct, trauma

### INTRODUCTION

Traumatic injuries to the porta hepatis are uncommon but are being recognised with increasing frequency [1,2]. These injuries are usually dramatic in their life-threatening potential but presentation and diagnosis can be delayed, particularly in cases of blunt abdominal trauma. This report highlights the problem of delayed presentation and the subsequent morbidity which can result.

### CASE REPORT

A healthy 73 year old female restrained driver was involved in a head-on collision. She sustained a fractured right radius and ulna, lacerations of the left lower leg and multiple bruises to the forehead and both upper limbs. Eighteen days later, she developed abdominal pain and hypotension and her haemoglobin dropped to 7g/dl. She was transfused with 4 units of packed cells but developed jaundice 3 days after transfusion with abnormal liver function tests (obstructive pattern). Ultrasound and CT scans of the abdomen revealed fluid in the peritoneal cavity with a localised collection in the region of the pancreas and a haematoma in the region of the caudate lobe. There was marked intrahepatic bile duct dilatation. Laparotomy revealed a haematoma contained within the peritoneum over the biliary confluence (hilar plate), with compression and obstruction of the bile ducts. In one area over the haematoma, the hilar plate had ruptured allowing bleeding into

*To whom correspondence addressed.

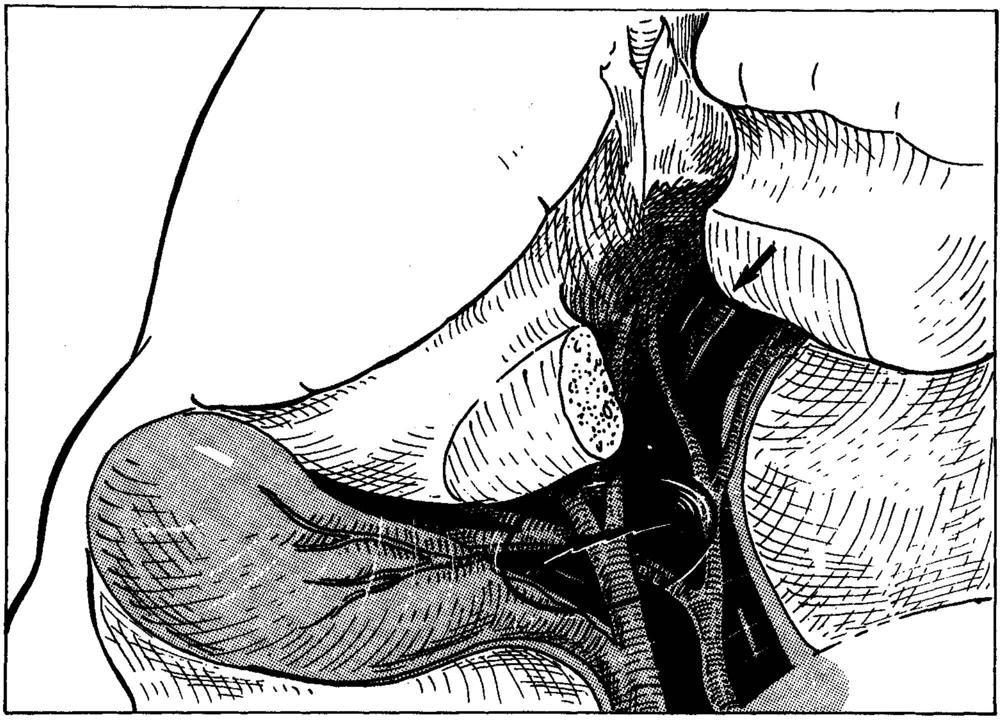

FIGURE 1 Illustration of the injury visualised after division of the parenchyma between segments IV and II. The falciform ligament was avulsed, producing a laceration of the left portal vein (arrow) and a haematoma which caused compression of the biliary confluence. Rupture through the hilar plate resulting in a haemoperitoneum had also occured.

the peritoneal cavity (Fig. 1). Following division of the parenchyma between segments IV and II and division of the hilar plate, the haematoma was evacuated. Bleeding was found to be due to avulsion of the falciform ligament from the left branch of the portal vein. Bleeding was controlled by a Pringle manoeuvre and the vein was sutured. Cholecystectomy was performed and a T-tube was inserted in the common bile duct. A feeding jejunostomy was inserted. Post-operative recovery was slow and she required ventilation for 4 days. Her jaundice resolved completely. A T-tube cholangiogram 7 days post-operatively was normal. She remained well at review, three months post-operatively.

### DISCUSSION

Traumatic injuries to the porta hepatis are uncommon. Since the first report by Drysdale in 1861 [3], the overall reported incidence at laparotomy for trauma is between 0.2 and 5% [1,4]. The largest series consists of 53 patients [5]. Injuries to the porta hepatis itself are usually not fatal. If death occurs it is usually due to associated liver or other serious injuries. Among the structures involved in porta hepatis injuries, portal vein injury is associated with the highest mortality, up to 50% in some series [2,4,5,6]. Most deaths are due to early uncontrolled haemorrhage.

The mechanism of injury usually involves penetrating trauma in the adult and blunt trauma in children. With the rising incidence in high speed motor vehicle accidents, blunt trauma is becoming more frequent. The common bile duct is the structure most commonly injured while the portal vein and hepatic artery are less frequently involved [1,2,6]. Isolated blunt injury to the portal vein, as occurred in this case, is very rare. Various factors have been suggested in the mechanism of blunt trauma to the porta hepatis.

These include an upward shearing force exerted by the liver on the relatively fixed bile ducts, the relative rigidity of the bile ducts and rapid emptying of the gallbladder due to increased intra-abdominal pressure [7]. The rarity of portal vein or hepatic artery injury is explained by their relatively higher elasticity and tortuosity and increased length. In view of this, it is surprising that in this case an isolated portal vein injury with no biliary injury was found.

Diagnosis of porta hepatis injury is difficult, especially in cases of blunt trauma. Associated injuries may distract the attention of the surgeon. In addition, injuries resulting from blunt trauma may present late or with an unexpected clinical picture. These problems were highlighted in this case. Presumably, the mechanism of injury in this case involved avulsion of the left portal vein at the time of injury with bleeding which was initially controlled but later became profuse, resulting in a haematoma and rupture through the hilar plate. Even in penetrating injuries, occult porta hepatis injuries can be missed at laparotomy. A high index of suspicion is essential if the diagnosis is not to be missed and subsequent morbidity and mortality avoided. A pre-operative peritoneal lavage may be helpful especially if bile is detected [2,4], although this is rarely the case. Intra-operative indicators of a porta hepatis injury include bile leak or staining, subseroal haematoma or profuse bleeding from the portal vein or hepatic artery.

The management of these injuries remains a challenge to surgeons. While some controversy exists, there is a developing trend in the recent approaches to a traumatic injuries of porta hepatis. In portal vein injuries, uncontrollable haemorrhage and sequelae of profound shock account for the majority of deaths. Therefore, rapid control of haemorrhage is essential. We employed the Pringle manoeuvre in this case which was highly effective. The use of a vascular clamp on the portal vein or a Fogarty balloon catheter may be necessary in some cases. After identifying the site of injury and obtaining adequate exposure by dissection of the hilar plate, various methods of repair may be employed. These include lateral venorrhaphy, end-to-end anastomosis, vein graft interposition, portacaval shunt or ligation of the portal vein. The combined experience from reported literature shows that lateral venorrhaphy resulted in the lowest mortality rate while the outcome following ligation was poor [4,6,8,9]. Portacaval shunt and ligation should be avoided and be used only as a last resort when other methods are not possible. In isolated hepatic artery injuries, simple ligation is usually of no consequence provided that there is no pre-existing liver disease or cirrhosis in which the liver relies on increased arterial flow [6]. Portal vein oxygen delivery is usually adequate in the normal liver [4]. Primary repair of incomplete hepatic arterial transection is an occasional option. In patients with injuries to both the hepatic artery and portal vein, repair of at least one is mandatory, preferably the portal vein. In such cases, the risk of liver ischaemia and subsequent necrosis is significantly increased [6,9].

In summary, traumatic injuries to porta hepatis are rare and presentation and diagnosis are often delayed. The diagnosis and management of the condition require a high index of suspicion and surgical expertise. With accurate diagnosis and prompt surgical treatment, the morbidity and mortality could be reduced.

### *References*

[1] Posner, M.C. and Moore, E.E. (1985). Extrahepatic biliary tract injury: operative management plan. *J Trauma*, **25**, 833–837.

[2] Kitahama, A., Elliott, L.F., Overby, J.L. and Webb, W.R (1982).The extrahepatic biliary tract Injury: perspectives in diagnosis and treatment. *Ann Surg.*, **196**, 536–541.

[3] Drysdale, T.M. (1861). Case of rupture of the common duct of the liver. Formation of a cyst containing bile. Death occurring on the fifty-third day. Autopsy. *Am J Med Sci.*, **12**, 399–404.

[4] Dawson, D.L., Johansen, K.H. and Jurkovich, G.J. (1991). Injuries to the portal triad. *Am J Surg.*, **161**(5), 545–551.

[5] Bade, P.G., Thomson, S.R., Hirshberg, A. and Robbs, J.V. (1989). Surgical options in traumatic injury to the extrahepatic biliary tract. *Br J Surge.*, **76**, 256–258.

[6] Busuttil, R.W., Kitahama, A., Cerise, E., McFadden, M., Lo, R. and Longmire, W.P. (1980). Management of blunt and penetrating injuries to the porta hepatis. *Ann Surg.*, **191**(5), 641–647.

[7] Feliciano, D.V., Bitondo, C.G., Burch, J.M., Mattox, K.L., Beall, A.C. and Jordan, G.L. (1985). Management of traumatic injuries to the extrahepatic biliary ducts. *Am J Surg.*, **150**, 705–709.

[8] Ivatury, R.R., Nallathambi, M., Lankin, D.H., Wapnir, I. Rohman, M. and Stahl, W.M. (1987). Portal vein injuries. Noninvasive follow-up of venorrhaphy. *Ann Surg.*, **206**(6), 733–737.

[9] Petersen, S.R. Sheldon, G.F. and Lim, R.C. (1979). Management of portal vein injuries. *J Trauma.*, **19**, 616–620.

